# Wanting this, not that: The neural circuit that turns specific expectations into actions

**DOI:** 10.1371/journal.pbio.3003859

**Published:** 2026-07-10

**Authors:** Joey A. Charbonneau, Erin L. Rich

**Affiliations:** Center for Neural Science, New York University, New York, New York, United States of America

## Abstract

Neuroeconomics has long focused on reward values, ignoring their identity. This Primer explores a new study in PLOS Biology that shows that identity-specific reward expectations in lateral orbitofrontal cortex steer goal-directed choices through a motivational circuit involving nucleus accumbens.

## Teaser

Neuroeconomics has long focused on reward values, ignoring their identity. A new *PLOS Biology* study shows that identity-specific reward expectations in the lateral orbitofrontal cortex steer goal-directed choices through a motivational circuit involving the nucleus accumbens.

“We have food at home.” Every child knows this response, delivered from the driver’s seat as the car glides past a favorite burger joint or pizza place approaching dinner time. The parental logic is hard to contest: a meal at home is just as filling, just as affordable, and perhaps just as tasty. On paper, the options are equivalent. However, the craving stirred by a glimpse of the restaurant logo is not for food in general—it is for that specific meal. Regardless of nutritional, volumetric, financial, or other equivalence, alternatives do not match the expectation once it has been formed. This everyday experience points to something that theories of decision-making have largely overlooked: the specific identity of a reward. What a reward is—above and beyond what it is worth—can be a powerful driver of behavior on its own. A study recently published in PLOS Biology by Witkowski and colleagues [1] asks how these expectations are represented in the brain, and in doing so reveals a neural pathway that translates identity-specific expectations into goal-directed choices.

The dominant framework in decision neuroscience treats rewards as interchangeable bundles of abstract value. Under this view, the brain sizes up each option, assigns it a number, and probabilistically picks the biggest one. The orbitofrontal cortex (OFC) has long been the leading candidate for where these computations happen, with decades of evidence showing that its activity tracks how desirable a reward is, across species and across reward types [2,3]. The appealing feature of this account is its simplicity: a single common currency lets the brain compare anything to anything. However, when you drive past the restaurant, the expectation that surfaces is not an abstract utility score, but rather something much more specific—a taste, a smell, an image. A growing body of work has found that the lateral OFC (lOFC) does more than track abstract value. lOFC appears to hold a detailed anticipatory model of what a specific reward will be, distinct from other rewards that may be equally pleasant [4–6]. When lOFC is damaged, animals can no longer adjust when circumstances change—they keep pursuing a reward that has been made less pleasant, as though the expectation driving their behavior has become untethered from its current value [7]. This suggests that the brain is likely maintaining rich, identity-specific models of expected rewards, and that these models, not just a simple value score, guide our actions.

Prior work in animals has established that identity-specific anticipation has real behavioral consequences. When a cue predicting a particular reward is present, animals preferentially pursue actions that earn that specific reward rather than other rewards of equal value [8]. This effect depends on lOFC working in concert with a motivational region called the nucleus accumbens [9]. However, whether these same principles apply in humans, and whether lOFC identity signals actively determine which option a person chooses at the moment of a decision, has not previously been directly tested.

Witkowski and colleagues took on this question in a human brain imaging study designed to pit identity-specific expectations against each other at the moment of choice [1]. Participants first learned that certain visual cues each predicted a specific food odor, then made choices between options associated with those odors. Before each choice, a pair of cues appeared—sometimes both pointing to the same reward, sometimes pulling toward different ones. The key finding was that lOFC activity in the moments before the choice reflected which specific reward the person was anticipating from the cue. When two competing expectations were evoked simultaneously, whichever was stronger in lOFC tended to win at the time of decision. Crucially, this effect could not be explained by individual differences in how pleasant participants found each odor because the identity of the anticipated reward was doing the work, not its value. The region of lOFC that tracked anticipatory identity was also the same region in which signals predicted the upcoming choice, making the case that these mental representations of specific rewards do not just reflect preference, but actively shape it.

**Fig 1:**
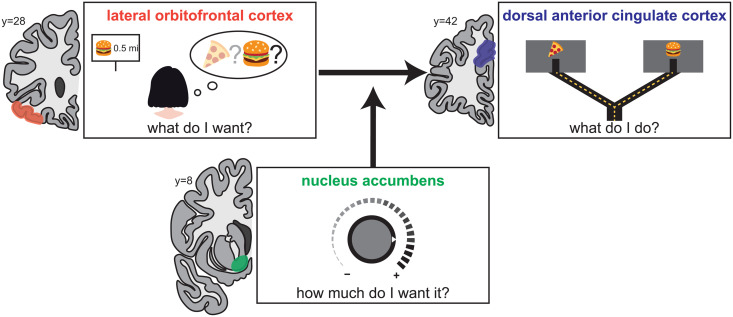
Neural circuitry linking identity-specific expectations to action. When a reward-predictive cue is encountered (here, a billboard), the lateral orbitofrontal cortex (lOFC; red) generates an expectation about the specific identity of the anticipated reward. The strength of this identity-specific expectation predicts which reward a person will subsequently choose to pursue when two possible rewards have equal value. The nucleus accumbens (NAc; green) appears to act as a motivational amplifier, controlling how strongly the lOFC identity signal drives action selection in the dorsal anterior cingulate cortex (dACC; blue), which encodes the action required to obtain the anticipated reward. When NAc activity is high, lOFC identity expectations are strongly coupled to dACC action representations, but when it is low, this coupling is absent.

Knowing what you want is one thing; acting on it is another. Witkowski and colleagues further went on to answer the question of how an identity-specific expectation in the brain gets converted into a goal-directed action (Fig 1). The answer, it turns out, involves a key motivational link in the chain. A brain region involved in converting reward-predictive cues into motivated behavior, the nucleus accumbens, proved to be critical. People whose brains responded more strongly to the identity-predicting cues in this region showed a tighter connection between what they were expecting and what they chose to do. More strikingly, this motivational signal appeared to relay the expectation forward to areas that directly plan and select actions. When motivational drive was high, the anticipated reward was reflected in the brain’s action planning signals. When motivational drive was low, the link was absent. The result is a picture of how cues like a billboard or a familiar smell can reach all the way from an expectation to motivated behavior. A specific expectation is formed, motivational systems amplify it, and that amplified signal shapes what actions we take.

The findings of Witkowski and colleagues matter for how we think about decision-making more broadly. The standard value-based account predicts that two equally pleasant options should be equally likely to be chosen [2], but this study shows that this is not always true. Specific rewards that are more vividly anticipated in the moment of choice might tip the balance, regardless of value. Specificity also makes the system adaptable. A reward expectation that carries real content—this food, this taste—can be evaluated against your current state and context in ways that an abstract value signal may not be able to [10]. The same cue that drives you toward pizza when hungry might lose its pull the moment you remember you had it for lunch. This machinery may also explain why some cravings are so difficult to resist. Compulsive behavior in addiction or binge eating, for instance, is unlikely to result from an excess of desire in general, but the pull of one particular thing, the identity of which is vividly reinstated by a cue [11]. This is a pull that value alone cannot override. Future studies might examine whether the circuit described here behaves differently in people who struggle with this type of impulsive, identity-specific craving, and whether targeting this system therapeutically could help override aberrant expectations and regain control over cue-driven behavior.
